# SeBioGraph: Semi-supervised Deep Learning for the Graph via Sustainable Knowledge Transfer

**DOI:** 10.3389/fnbot.2021.665055

**Published:** 2021-04-01

**Authors:** Yugang Ma, Qing Li, Nan Hu, Lili Li

**Affiliations:** ^1^School of Architecture and Urban Planning, Chongqing University, Chongqing, China; ^2^School of Computer Science, Northwestern Polytechnical University, Shaanxi, China; ^3^School of Management Science and Real Estate, Chongqing University, Chongqing, China; ^4^China Construction Science & Technology Group Co., Ltd. Shenzhen, China; ^5^College of Civil and Environmental Engineering, Harbin Institute of Technology, Harbin, China

**Keywords:** graph, semi-supervised deep learning, knowledge transfer, link prediction, node classification

## Abstract

Semi-supervised deep learning for the biomedical graph and advanced manufacturing graph is rapidly becoming an important topic in both academia and industry. Many existing types of research focus on semi-supervised link prediction and node classification, as well as the application of these methods in sustainable development and advanced manufacturing. To date, most manufacturing graph neural networks are mainly evaluated on social and information networks, which improve the quality of network representation y integrating neighbor node descriptions. However, previous methods have not yet been comprehensively studied on biomedical networks. Traditional techniques fail to achieve satisfying results, especially when labeled nodes are deficient in number. In this paper, a new semi-supervised deep learning method for the biomedical graph via sustainable knowledge transfer called SeBioGraph is proposed. In SeBioGraph, both node embedding and graph-specific prototype embedding are utilized as transferable metric space characterized. By incorporating prior knowledge learned from auxiliary graphs, SeBioGraph further promotes the performance of the target graph. Experimental results on the two-class node classification tasks and three-class link prediction tasks demonstrate that the SeBioGraph realizes state-of-the-art results. Finally, the method is thoroughly evaluated.

## Introduction

Graph analysis can be used for various fields including linguistics (Akimushkin et al., 2017), social sciences (Rozemberczki et al., 2019), and biology (Theocharidis et al., 2009; Subramani et al., 2015). In biomedical graphics, the modeling of entities and their relations is indispensable for different tasks. Specifically, discovering synergistic or antagonistic effects between multiple drugs through drug-drug interaction graphs (Segura-Bedmar et al., 2011), developing new drugs for the disease through drug-disease graphs (Zhu Q. et al., 2013), and assisting doctors in clinical decision-making via disease-symptom graphs are some typical task scenarios (Li et al., 2019).

Biological graphs are notoriously complex and hard to decipher. Until now, many biomedical graph analytic methods have been proposed to analyze it (Grover and Leskovec, 2016; Fan et al., 2018; Zhang et al., 2018b). Most of these approaches transform the original data into vectorial data. In addition, the representation of the network is updated by integrating neighbor node descriptions. Therefore, the structure information of the graph is preserved by the low-dimension representation of nodes. The various downstream tasks of the biomedical graph can be divided into three categories, as follow: clustering, link prediction, and node classification (Hamilton et al., 2017; Cai et al., 2018). Among them, the clustering analytic task aims to capture subsets of approximate nodes and then collect them together. The link prediction task is referred to predicting possible links or missing links. The node classification task is to determine the label of nodes.

However, these state-of-the-art graph analytic approaches are mainly evaluated on non-biomedical datasets. At the same time, most biomedical image analysis methods have limited receptive fields and only focus on shallow layers. These methods cannot perform medical traceability analysis. Especially, it becomes even more difficult to obtain satisfactory performance when the quantities of labeled nodes are scarce. Prediction of a link or classifying a node has been challenging, because manual annotations are often expensive, only a few nodes are involved. Most human-labeled biomedical graph features are always insufficient, while machine-labeled biomedical graph features are not sufficient to characterize entities. All these lead to the inability to build reliable and effective models. It follows that it is even more challenging to achieve semi-supervised deep learning for on biomedical graph than on independent identically distributed data (e.g., biomedical images).

More comparison details can be found in Table 1.

**Table 1:** A summary of 12 representative graph methods and existing work using them for a biomedical graph task.

**Model**	**Node classification tasks**		**Link prediction tasks**			
	**Medical term type classification**	**Protein function prediction**	**Drug-disease association prediction**	**Drug-drug interaction prediction**	**Protein-protein interaction prediction**	**Chemical-protein interaction prediction**
**Matrix factorization**						
Singular Value Decomposition (De Lathauwer et al., 2000)	N	Y (Cho et al., 2016)	Y (Dai et al., 2015)	N	Y (You et al., 2017)	N
Locally Linear Embedding (Roweis and Saul, 2000)	N	N	N	N	N	Y (Pliakos et al., 2019)
Laplacian (Belkin and Niyogi, 2003)	N	Y (Fan et al., 2018)	Y (Zhang et al., 2018b)	Y (Zhang et al., 2018a)	Y (Zhu L. et al., 2013)	N
GF (Ahmed et al., 2013)	N	N	Y (Yang et al., 2014; Zhang et al., 2018b)	Y (Zhang et al., 2018a)	N	N
GraRep (Cao et al., 2015)	N	N	N	N	N	N
HOPE (Ou et al., 2016)	N	N	N	N	N	N
**Random walk**						
DeepWalk (Perozzi et al., 2014)	N	Y (Cho et al., 2016; Kulmanov et al., 2018)	N	N	N	N
node2vec (Grover and Leskovec, 2016)	N	Y (Grover and Leskovec, 2016; Zitnik and Leskovec, 2017)	N	N	N	N
struc2vec (Ribeiro et al., 2017)	N	N	N	N	N	N
**Neural network**						
LINE (Tang et al., 2015)	N	N	N	N	N	N
GAE (Tang et al., 2016)	N	N	N	Y (Ma et al., 2018; Zitnik et al., 2018)	N	N
SDNE (Wang et al., 2016)	N	Y (Gligorijevic et al., 2018)	N	N	Y (Wang et al., 2017)	N

### Matrix Factorization

Matrix factorization technology has been broadly utilized for graph data analysis areas, including but not limited to social networks, natural language processing, and computer vision. Through matrix factorization, different kinds of the graph can be presented as affinity. Besides, each vertex can be represented via a low-dimensional vector. Both Locally Linear Embedding (LLE) (Roweis and Saul, 2000) and Singular Value Decomposition (SVD) (De Lathauwer et al., 2000) are first focus on factorizing the 1st-order data matrix. And then, the method developed Laplacian Eigenmaps (LE) (Belkin and Niyogi, 2003) and Graph Factorization (GF) (Ahmed et al., 2013).

Due to the limitation of representation, researchers attempt to retain the graph structure by constructing various high order data proximity matrices, such as GraRep and HOPE. GraRep (Cao et al., 2015) proposes using k-step transition probability matrices to factorization. At the same time, it optimized through stochastic gradient descent, but it only applies to undirected graphs. HOPE (Ou et al., 2016) adopts network similarity measures to preserve high order network frameworks.

### Random Walk

To a specified starting node and corresponding graph, the random walk approach choosees an adjacent node randomly and walk to this node. Generally, if the graph is too small or too large, this method is particularly useful to measure the graph completely.

DeepWalk (Perozzi et al., 2014) is a recently proposed method, which only suits social graphs with binary edges. In DeepWalk, random walks are mainly adopted to enlarge the neighbor of every vertex. However, it fails to provide a clear goal that definitely expresses which graph properties are retained. At the same time, it only applies to the un-weighted graph. Similarly, Node2vec (Grover and Leskovec, 2016) reserves the higher-order proximity between various nodes. The node2vec uses a biased random walk. It can balance the depth-first and breadth-first search, so it can get more graph information than DeepWalk. Additionally, Struc2vec (Ribeiro et al., 2017) first utilizes a hierarchy weighted graph to encode the similarity between nodes. In this structure, each layer k is decided by the k-hop neighbor nodes.

### Graph Neural Networks

Recently, GNNs are broadly adopted for data analysis (Kipf and Welling, 2016; Ravi and Larochelle, 2016; Finn et al., 2017; Huang et al., 2019; Liu et al., 2019; Zhang et al., 2019; Tang et al., 2020). It aims to encode the nodes with signals that lie in the receptive fields (Kipf and Welling, 2016). There are three lines of GNNs methods: non-supervised methods, semi-supervised methods, and supervised methods. All of these three approaches have gained great breakthroughs in diverse graph-based tasks, such as graph classification and node classification. However, these progressive methods are most analyzed and evaluated on non-biomedical graphs (e.g., social graphs) (Tang et al., 2015, 2016; Wang et al., 2016; Velickovic et al., 2017). Therefore, only a few studies have targeted biomedical networks (Wang et al., 2017; Gligorijevic et al., 2018; Ma et al., 2018; Zitnik et al., 2018).

In LINE (Finlayson et al., 2014), two functions are defined which include a 1st-order and a 2nd-order proximities function. And then, it minimizes the combination of the two functions. The first-order proximity function is much the same as that of the GF model (Ahmed et al., 2013). However, the LINE differs in that there are two joint probability distributions for each vertices pair, one using the embedding and the other using the adjacency matrix. GAE (Tang et al., 2016) input an adjacency matrix that relies on graph convolutional network encoder to obtain the higher-order dependencies of nodes. They have proved that the use of variational autoencoders can promote performance. Structural Deep Network Embedding (SDNE) (Wang et al., 2016) adopts auto-encoders to embedding graph nodes and acquire highly non-linear dependencies. In this model, there are two portions including supervised and unsupervised. For the first supervised portion, it imposes punishment when similar vertices are projected too far away from each other in the vector space. For the latter, it is equivalent to an auto-encoder and aims to find a representation for each node that can regenerate its neighbor.

We adopt a biomedical graphs analytic method that which has both excellent performance and enhanced interpretability. We are proposed to leverage the prior knowledge acquired from auxiliary graphs to enhance the performance of the target graphs. In addition to local topological structures, the auxiliary graphs and target graphs may share class-dependent node features. For this purpose, we proposed SeBioGraph, a new semi-supervised deep learning method for the biomedical graphs via knowledge transfer. Base on semi-supervised metric few-shot learning, the SeBioGraph intends to learn a transferable metric space, which predicts the label of each node through the class of the closest prototype to the node. It aims to optimize this mapping so that geometric relationships in the metric space reflect the structure of the original biomedical graphs. The metric space is to combine two parts: the embedded node and the prototype of each class.

The construction of SeBioGraph consists of the following steps. At first, a graph encoder, which is mainly Graph Neural Networks (GNNs) (Kipf and Welling, 2016), is utilized to learn the information of every node. Accordingly, multiple node features (e.g., disease feature, drug chemical substructure features, and target protein feature) are mapped into a common subspace. In this subspace, it maintains the immutability of the original indication labels of nodes. Then, to obtain biomedical graphs' global information sufficiently, we construct a relational framework for all identical category samples. Through the embedding function of these two types of encrypted structured knowledge, the problem of lack of labeled nodes is compensated. After that, we design hierarchical biomedical graph representations gate to emphasize the analogous biomedical graphs having close metric spaces. Finally, in order to enhance the quality of node representation and robustness of training, we design an auxiliary graph constraint.

To sum up, our contributions can be outlined as follows:

- To the best of our awareness, it is the pioneering work to successfully perform the sustainable knowledge transfer to improve semi-supervised deep learning for the biomedical graphs;
- We propose a novel SeBioGraph to address the issue, which can simultaneously transfer all-graph-level and part-node-level structures across different graphs;
- SeBioGraph outperforms baseline models in two benchmark datasets in node classification tasks and five biomedical link prediction tasks, showing its potential to serve as an effective general-purpose representation learning algorithm for biomedical graph data.

## Methodology

**Figure 1:**
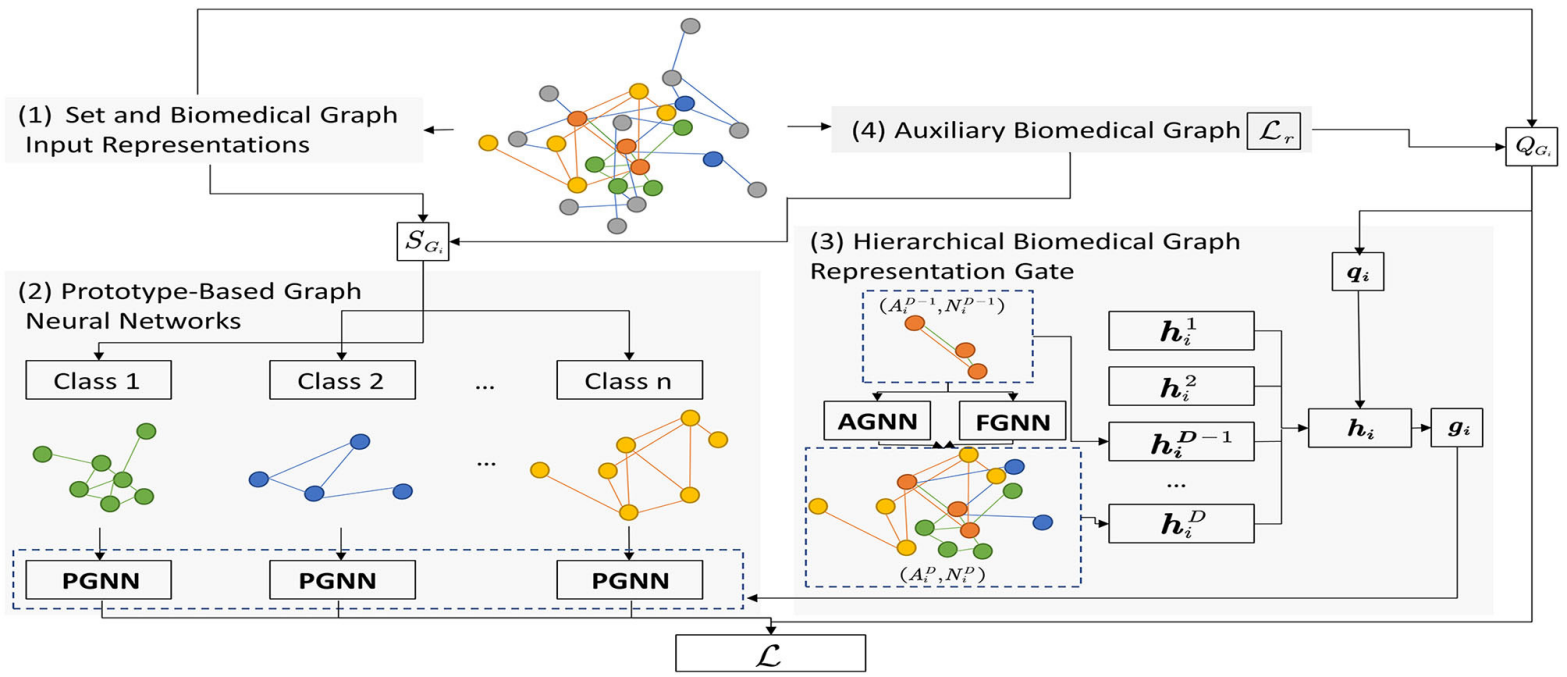
The overall framework of SeBioGraph.

In this part, we introduce our proposed method SeBioGraph detailed. An illustration of the framework is shown in Figure 1. Here, we describe four parts of the proposed structure: set and biomedical graph input representations, prototype-based graph neural networks, hierarchical biomedical graph representation gate, and auxiliary biomedical graph.

### Set and Biomedical Graph Input Representations

The input biomedical graph neural networks *G* = (*A, N*) contain a collection of links and nodes, where is *A* ∈ {0, 1}^*m*×*m*^ the adjacent matrix, and $N=\left\{n_{1},...,n_{m}\right\}\in R^{m\times h}$ is the node feature matrix. We set a batch of graphs {*G*_1_, ..., *G*_*N*_*t*__} sampled from a probability distribution ^ε^.

Each node has two different functions in a biomedical graph: first is local interactions with different classes of neighbors; second is the same classes of neighbors. For example, (a) the structure between drug-disease nodes describing their co-association, as well as the structure between chemical-protein nodes describing their co-interaction, (b) the local interactions between protein nodes, chemical nodes, disease nodes, and drug nodes. So we will use *S*_i_ to denote a support-nodes set and *Q*_i_ to denote a query-nodes set, where:

(1) $$\begin{array}{l} S_{i}={\left\{\left({\text{n}}_{i,j}^{s_{i}},y_{i,j}^{s_{i}}\right)\right\}}_{j=1}^{m^{s_{i}}} \end{array}$$

(2) $$\begin{array}{l} Q_{i}={\left\{\left({\text{n}}_{i,j}^{q_{i}},y_{i,j}^{q_{i}}\right)\right\}}_{j=1}^{m^{q_{i}}} \end{array}$$

Where $m^{S_{i}}$ is a small set of labeled, and *y*_*i,j*_ ∈ {1, ...*K*} is the corresponding label.

In graph *G*_*i*_, the effectiveness on *Q*_*i*_ is evaluated by the loss function *L*_*i*_ for every node*j*, as shown in Equation (3). where $\left|S_{i}^{k^{\prime }}\right|$ is the number of samples in *S*_*i*_. of class *k*, and $Q_{i}^{k}$ denotes the sample set in *Q*_*i*_ of class *k*. We then predict its relevant label by jointing its embedding $f_{\theta }\left(A,n_{i,j}^{q_{i}}\right):R^{h}\to R^{h^{\prime }}$ with representation $\left(f_{\theta }\left(A,n_{i,j}^{s_{i}}\right),y_{i,j}^{s_{i}}\right)$ in support nodes set *S*_*i*_ through the similarity *d*.

### Prototype-Based Graph Neural Networks

For each node in graph, the relation structure of the samples set belonging to class is extracted firstly. It is constructed based on similarity metrics (e.g., the inverse topological distance between nodes and the number of k-hop common neighbors). We denote the graph neural networks structured prototype as:

(3) $$\begin{array}{lll} L_{i} & = & -\underset{k}{\sum }\underset{\left({\text{n}}_{i,j},y_{i,j}^{q_{i}}\right)\in Q_{i}^{k}}{\sum }\log\frac{\exp\left(-d\left(f_{\theta }\left(\text{A},{\text{n}}_{i,j}^{q_{i}}\right),\underset{{\text{n}}_{i,j}^{s}\in S_{i}^{k}}{\sum }f_{\theta }\left(\text{A},{\text{n}}_{i,j}^{s_{i}}\right)/|S_{i}^{k}|\right)\right)}{\underset{k^{\prime }}{\sum }\exp\left(-d\left(f_{\theta }\left(\text{A},{\text{n}}_{i,j}^{q_{i}}\right),\underset{{\text{n}}_{i,j}^{\frac{y}{i}}\in S_{i}^{k^{\prime }}}{\sum }\left(f_{\theta }\left(\text{A},{\text{n}}_{i,j}^{x_{i}}\right),y_{i,j}^{s_{i}}\right)/|S_{i}^{k^{\prime }}|\right)\right)}\; \end{array}$$

(4) $$\begin{array}{lll} {\text{c}}_{i}^{k} & = & {\text{MaxPooling}}_{j=1}^{m_{i}^{k}}\left({\mathrm{PGNN}}_{\alpha }\left(D_{i}^{k},f_{\theta }\left(S_{i}^{k}\right)\right)\left[j\right]\right)\; \end{array}$$

Where $m^{S_{i}^{k}}$ is the number of nodes in $S_{i}^{k}$,$PGNN_{\alpha }\left(D_{i}^{k},f_{\theta }\left(S_{i}^{k}\right)\right)$ is the representation matrix in *j* − *th* node.

The globally shared parameter α of the PGNN is defined as a gate function *g*_*i*_ (more detail in section Results and Discussion) is defined as:

(5) $$\begin{array}{l} \alpha_{i}={{\text{g}}_{i}}^{○}\alpha =\sigma {\left({\text{W}}_{g}{\text{h}}_{i}+{\text{b}}_{g}\right)}^{○}\alpha \end{array}$$

Where ○ represents element-wise multiplication, *W*_*g*_ is a learnable weight parameter, and *b*_*g*_ is a learnable bias parameter.

### Hierarchical Biomedical Graph Representation Gate

In order to show the different topologies specific to the graph, we following the popular method of hierarchical graph modeling (Ying et al., 2018). Compare the PGNN with globally shared parameters ^α^, and the hierarchical biomedical graph representation gate combines two-level detail. There are biomedical graph node assignment and representation fusion.

#### Biomedical Graph Node Assignment

In this step, each low-level node *k*^*d*^ (in*d* − *th*level) is assigned to high-level node *k*^*d*+1^community. The biomedical graph node assignment value is calculated by applying a softmax function, which is defined as follows:

(6) $$\begin{array}{l} p_{i}^{k^{d}\to k^{d+1}}=\frac{\exp\left(AGNN\left({\text{A}}_{i}^{d},{\text{N}}_{i}^{d}\right)\left[k^{d},k^{d+1}\right]\right)}{\overset{K+1}{\underset{k^{d+1}=1}{\sum }}\exp\left(AGNN\left({\text{A}}_{i}^{d},{\text{N}}_{i}^{d}\right)\left[k^{d},k^{d+1}\right]\right)} \end{array}$$

where AGNN is the assigned value of the biomedical graph node, which is from the node *k*^*d*^ in the bottom layer *d* to the node *k*^*d*+1^ in the high layer *d* + 1, the $AGNN\left(A_{i}^{d},N_{i}^{d}\right)\left[k^{d},k^{d+1}\right]\in R^{1}$. So we could be getting the biomedical graph node assignment matrix $P_{i}^{K^{d}\to K^{d+1}}\in R^{K^{d}\times K^{d+1}}$. It includes each level of biomedical graph node assignment value $p_{i}^{k^{d}\to k^{d+1}}$.

#### Representation Fusion

For level *d* + 1, the adjacent matrix $A_{i}^{d+1}$ and the node feature matrix $N_{i}^{d+1}$ are defined as follows:

(7) $$\begin{array}{lll} {\text{A}}_{i}^{d+1} & = & {\left({\text{P}}_{i}^{d\to d+1}\right)}^{T}{\text{A}}_{i}^{d}{\text{P}}_{i}^{d\to d+1} \end{array}$$

(8) $$\begin{array}{lll} {\text{N}}_{i}^{d+1} & = & {\left({\text{P}}_{i}^{d\to d+1}\right)}^{T}FGNN\left({\text{A}}_{i}^{d},{\text{N}}_{i}^{d}\right) \end{array}$$

where FGNN is the fusion GNN. Then, the feature representation $h_{i}^{d+1}$ can be obtained through jointing the information of all nodes, which is defined as follows:

(9) $$\begin{array}{l} {\text{h}}_{i}^{d+1}={\mathrm{MaxPooling}}_{k^{d}+1=1}^{K^{d+1}}\left({\left({\text{P}}_{i}^{d\to d+1}\right)}^{T}FGNN\left({\text{A}}_{i}^{d},{\text{N}}_{i}^{d}\right)\left[k^{d+1}\right]\right) \end{array}$$

So we could be getting the biomedical graph structure representation set $\;\left\{{\text{h}}_{\text{i}}^{\text{1}}\text{,}...{\text{,h}}_{\text{i}}^{\text{D}}\;\right\}\;$ from varied levels. After that, the overall biomedical graph structure representation *h*_*i*_ is represented by the aggregator *AGG* of each level. We use attention aggregators to represent different levels of contributions to the whole representation, which is defined as:

(10) $$\begin{array}{l} {\text{h}}_{i}=\mathrm{AttAGG}\left(\left\{{\text{h}}_{i}^{1},\ldots ,{\text{h}}_{i}^{D}\right\}\right)=\overset{D}{\underset{d=1}{\sum }}\frac{{\text{q}}_{i}^{T}{\text{h}}_{i}^{d}}{\overset{D}{\underset{d^{\prime }=1}{\sum }}{\text{q}}_{i}^{T}{\text{h}}_{i}^{d^{\prime }}}{\text{h}}_{i}^{d} \end{array}$$

Where *q*_*i*_ is a learnable query vector.

The biomedical graph representation gate *g*_*i*_ maps the specific graph representation *h*_*i*_ to the identical space of parameter α_*i*_ as follow:

(11) $$\begin{array}{l} {\text{g}}_{i}=T\left({\text{h}}_{i}\right)=\sigma \left({\text{W}}_{g}{\text{h}}_{i}+{\text{b}}_{g}\right) \end{array}$$

Thus, Equation (5) would be updated.

### Auxiliary Biomedical Graph

Graph semi-supervised deep learning aims to learn a well-generalized embedding function from previous graphs. This function can be used to a new graph with a small support set. At the same time, we need to design a new constraint loss function to optimize the training robustness and the quality of node embedding.

(12) $$\begin{array}{l} L_{d}\left({\text{A}}_{i},{\text{N}}_{i}\right)={\left\|{\text{A}}_{i}-GNN_{dec}\left({\text{A}}_{i},{\text{H}}_{i}\right)GNN_{dec}^{T}\left({\text{A}}_{i},{\text{H}}_{i}\right)\right\|}_{F}^{2} \end{array}$$

Where ‖·‖ *F* represents the Frobenius norm.

In the end, the optimization problem of SeBioGraph is defined as follows:

(13) $$\begin{array}{l} \mathrm{Min}\Phi \leftarrow \Phi -\gamma \nabla_{\Phi }\overset{N_{t}}{\underset{i=1}{\sum }}L_{i}\left({\text{A}}_{i},{\text{N}}_{i}\right)+\beta L_{d}\left({\text{A}}_{i},{\text{N}}_{i}\right) \end{array}$$

where Φ represents all learnable parameters.

## Experiments

### Tasks and Dataset

In this section, we evaluate the quality of SeBioGraph for two-class biomedical graph tasks in eight datasets. The first-class tasks are node classification, i.e., protein-protein interaction with functional annotations and semantic type classification of medical term. The second-class tasks are link prediction, i.e., chemical-disease interaction prediction, drug-drug interaction prediction, chemical-protein interaction prediction.

#### Node Classification Tasks

The task of node classification is a very important first step of graph analysis. For a partly labeled graph, this task is to predict the class of unlabeled nodes. In 2018, Gligorijevic proposed to obtain the representation of proteins via developing deepNF models (Gligorijevic et al., 2018). In the same year, Lim adopts a method based on regularized Laplacian kernel, which can learn the low-dimensional graph feature of proteins (Fan et al., 2018). To evaluate the impact of semi-supervised deep learning biomedical graphs, we use classification tasks based on a single unlabeled node. Here, SeBioGraph focused on the following two kinds of node classification tasks benchmark experimental datasets.

##### Medical Term Semantic Type Classification

We utilize a set of medical terms that can be obtained publicly and their co-occurrence statistics datasets (Clin Term COOC) (Finlayson et al., 2014). For two terms, we compute its co-occurrence frequencies based on 1-day. Besides, we only save those edges whose PPMI is greater than two. The Clin Term COOC datasets contain 48,651 nodes.

##### Protein-Protein Interaction (PPI) With Functional Annotations

There are two PPI graphs datasets containing functional annotations, which are node2vec and MashUp. The first one is Node2vec (Grover and Leskovec, 2016), and it contains the 3,890 proteins node. The second one is MashUp (Cho et al., 2016), which contains six individual PPI graphs. It contains 16,143 proteins node and 300,181 protein-protein interactions.

#### Link Prediction Tasks

In the biomedical field, the discovery of new links (a.k.a. association, interactions) is an important task. For a series of biomedical entities and links, the purpose of this task is to predict some other hidden interactions of entities. Most previous methods focus on establishing biological feature engineering, such as graph topological similarities (Hamilton et al., 2017) and chemical substructures (Liang et al., 2017). After that, the semi-supervised graph inference model or supervised deep learning methods are utilized to predict potential interactions. In order to compare the performance of our model with the previous model more comprehensively. To compare performance with previous models, SeBioGraph focused on the following five kinds of link prediction tasks benchmark experimental datasets.

##### Chemical-Disease Association (CDA) Prediction

The Comparative Toxicogenomics Database (CTD) (Davis et al., 2019) is a public biomedical graph based on literature, which manually labeled associations between gene products, chemicals, diseases, and so on. We filtered the association biomedical graph between 12,765 chemical-disease nodes in the CTD graph.

##### Drug-Disease Association (DDA) Prediction

The DDA prediction database is NDF-RT (National Drug File Reference Terminology) (Bodenreider, 2004) produced by the U.S. Department of Veterans Affairs. The drug characteristics are including related diseases, physiologic effects, and ingredients. We filtered the association biomedical graph between 13,545 drug-disease nodes in the NDF-RT graph.

##### Drug-Drug Interaction (DDI) Prediction

The DDI prediction database is DrugBank (Wishart et al., 2018), which contains detailed data about drugs including mechanisms, interactions and drug targets.

##### Protein-Protein Interaction (PPI) Prediction

The PPI prediction database is STRING (Szklarczyk et al., 2015), which includes indirect (functional) and direct (physical) associations. We filtered the association biomedical graph between 15,131 protein-protein nodes in the STRING graph.

##### Chemical-Protein Interaction (CPI) Prediction

The CPI prediction database is STITCH (Kuhn et al., 2007), which includes the interaction information of more than 68,000 different chemicals and 2,200 drugs. It links them to 1.5 million genes across 373 genomes. We filtered the association biomedical graph between 4,138,421 chemical-protein nodes in the STITCH graph.

### Experiments on the Parameter Settings

In these experiments, we use an open Python package of OpenNE to train the node representation in the SeBioGraph. For the link prediction tasks, our model is split the 80% for the training set and 20% for the testing set. In this work, we follow the traditional semi-supervised deep learning settings (Finn et al., 2017; Snell et al., 2017). The is a two-layer graph convolutional structure. In each layer, there are 32 neurons. For PGNN, AGNN, and FGNN, we adopt a one-layer graph convolutional structure as the substitute for GNN. Other weights are randomly initialized from a zero-mean Gaussian distribution. We tuned all the hyperparameters for our model 5-fold cross-validation for the optimization of the hyperparameters and report as final results.

### Results and Discussion

#### Node Classification Tasks

Table 2 illustrates the result of various biomedical graph analytic methods on protein function prediction and medical term semantic type classification task. We use two F1 weighted criteria including Micro-F1 and Macro-F1 to evaluate the performance of different approaches. For the Macro-F1, it computes metrics for every label type, and then acquires their un-weighted mean. For the Micro-F1, it computes metrics globally by counting all samples.

**Table 2:** Comparison between SeBioGraph and other node classification methods on three biomedical graph datasets.

**Method**	**Clin Term COOC**		**Node2vec PPI**		**MashUp PPI**	
	**Micro-F1**	**Macro-F1**	**Micro-F1**	**Macro-F1**	**Micro-F1**	**Macro-F1**
**Matrix factorization**						
SVD (De Lathauwer et al., 2000)	42.0 ± 0.5%	18.6 ± 0.7%	22.8 ± 1.1%	17.9 ± 1.1%	34.7 ± 1.4%	29.7 ± 1.4%
LLE (Roweis and Saul, 2000)	32.5 ± 0.7%	13.9 ± 0.4%	18.1 ± 0.9%	13.8 ± 1.2%	19.4 ± 1.3%	**37.5** ± **1.2%**
LE (Belkin and Niyogi, 2003)	31.3 ± 0.5%	7.3 ± 0.2%	10.1 ± 0.8%	7.0 ± 0.7%	13.2 ± 0.9%	10.7 ± 0.8%
GF (Ahmed et al., 2013)	35.2 ± 0.7%	14.3 ± 0.9%	16.8 ± 1.1%	12.1 ± 1.1%	29.0 ± 1.5%	23.7 ± 1.6%
GraRep (Cao et al., 2015)	42.4 ± 0.6%	17.7 ± 0.5%	23.8 ± 1.0%	19.3 ± 1.3%	33.4 ± 1.1%	28.3 ± 1.1%
HOPE (Cao et al., 2015)	39.5 ± 0.5%	16.3 ± 0.6%	20.8 ± 1.1%	15.2 ± 1.1%	32.2 ± 1.3%	26.6 ± 1.3%
**Random walk**						
DeepWalk (Perozzi et al., 2014)	47.2 ± 0.5%	22.7 ± 0.7%	24.3 ± 0.1%	19.4 ± 1.1%	35.7 ± 1.1%	31.1 ± 1.2%
Node2vec (Grover and Leskovec, 2016)	47.9 ± 0.5%	23.1 ± 1.0%	**24.3** **±** **0.9%**	19.0 ± 1.1%	36.7 ± 1.2%	31.3 ± 1.3%
Struc2vec (Ribeiro et al., 2017)	25.3 ± 0.6%	3.8 ± 0.1%	9.4 ± 0.6%	6.1 ± 0.4%	12.0 ± 1.0%	8.7 ± 0.8%
**Graph Neural networks**						
LINE (Tang et al., 2015)	45.3 ± 0.6%	20.5 ± 0.8%	23.6 ± 1.1%	17.6 ± 1.2%	35.2 ± 1.7%	29.6 ± 1.7%
GAE (Tang et al., 2016)	29.5 ± 1.2%	7.1 ± 0.7%	23.7 ± 1.4%	18.6 ± 1.4%	35.8 ± 1.3%	30.7 ± 1.4%
SDNE (Wang et al., 2016)	27.1 ± 1.6%	4.2 ± 0.7%	9.8 ± 1.0%	4.7 ± 0.7%	17.8 ± 1.3%	10.9 ± 1.2%
**Our model**						
SeBioGraph	**51.7** **±** **0.9%**	**24.3** **±** **1.0%**	23.6 ± 1.1%	**21.7** **±** **1.0%**	**42.4** **±** **1.4%**	35.4 ± 0.9%
- Auxiliary	46.5 ± 1.1%	19.2 ± 0.7%	21.9 ± 0.9%	19.9 ± 1.0%	36.8 ± 1.2%	31.5 ± 0.9%

*The meaning of bold is the best F1 precision*.

We divided the traditional methods into four groups: matrix factorization, random walk, graph neural networks, and our model. First, the matrix factorization methods used many features to the classifier, such as SVD, LLE, LE, GF, GraRep, and HOPE. According to the result, they achieved a Micro-F1 score of 42.4 ± 0.6% (GraRep) and a Macro-F1 score of 18.6 ± 0.7%. This shows that modeling the first-order proximity directly could be sufficient for basic classification nodes. The random walk model can catch more different functions for nodes in different subgraphs. The Node2vec performs better since it mostly pays attention to modeling the structural identity of each node. But the biomedical graph may not exist a clear structural role. Its accuracy is limited. The other model of graph neural network methods are an effective way for the node classification task. There are GNN-based models such as LINE (Tang et al., 2015), GAE (Tang et al., 2016), and SDNE (Wang et al., 2016). However, the graph neural network methods may have several flaws. On the one hand, it may be inaccurate. On the other hand, the parsing time will be exponentially increased by data. The last model is our model for SeBioGraph, which shows the advantage of prior knowledge obtained from the learned graphs. Experimental results show that our SeBioGraph reach an improvement of 1.2% on the Macro-F1 score and 3.8% on the Micro-F1 score. Obviously, it exceeds the second-best Node2vec.

To demonstrate the effect of each portion in SeBioGraph, the ablation experiments are implemented. By observing the results, we find that the auxiliary biomedical graph mechanism in SeBioGraph significantly outperforms Node2vec. Evidently, the auxiliary biomedical graph module plays an indispensable role in the experiment. Experimental results show that our model achieved a Micro-F1 score of 51.7 ± 0.9%, which performs better than other approaches. The auxiliary biomedical graph module enhances the performance by 5.2% than the model not applied it.

#### Link Prediction Tasks

For link prediction tasks, we comparison accuracy values on the five biomedical graph datasets: CTD CDA, NDF-RTDDA, DrugBank DDI, STRING PPI, and STITCH CPI. We report the averaged accuracy with 95% confidence intervals on the 10-shot classification in Table 3. It manifests the accuracy value generated for early prediction using graph neural networks, random walk and matrix factorization methods. The results attest that our SeBioGraph achieves a high accuracy value of 97.2 ± 0.5%, which excels all competing for state-of-the-art approaches.

**Table 3:** Comparison of accuracy value between SeBioGraph and other link prediction methods on five biomedical graph datasets.

**Method**	**CTD CDA**	**NDF-RT DDA**	**DrugBank DDI**	**STRING PPI**	**STITCH CPI**
**Matrix factorization**					
SVD (De Lathauwer et al., 2000)	93.6 ± 0.2%	77.9 ± 0.3%	91.9 ± 0.1%	86.7 ± 0.1%	31.7 ± 0.4%
LLE (Roweis and Saul, 2000)	86.5 ± 0.3%	89.7 ± 0.4%	89.1 ± 0.2%	79.8 ± 1.0%	29.4 ± 0.3%
LE (Belkin and Niyogi, 2003)	85.6 ± 0.4%	93.0 ± 0.3%	79.6 ± 0.2%	63.9 ± 2.1%	23.2 ± 0.5%
GF (Ahmed et al., 2013)	88.4 ± 0.4%	72.0 ± 0.6%	88.2 ± 0.3%	81.7 ± 0.5%	32.1 ± 0.3%
GraRep (Cao et al., 2015)	96.0 ± 0.1%	96.3 ± 0.1%	92.5 ± 0.1%	89.4 ± 0.1%	41.4 ± 0.4%
HOPE (Ou et al., 2016)	95.1 ± 0.1%	94.9 ± 0.1%	92.3 ± 0.1%	83.9 ± 0.1%	42.7 ± 0.2%
**Random walk**					
DeepWalk (Perozzi et al., 2014)	92.9 ± 0.2%	78.3 ± 0.4%	92.1 ± 0.1%	88.4 ± 0.1%	26.4 ± 0.3%
Node2vec (Grover and Leskovec, 2016)	91.1 ± 0.2%	81.9 ± 0.5%	90.2 ± 0.1%	82.8 ± 0.3%	37.7 ± 0.6%
Struc2vec (Ribeiro et al., 2017)	96.5 ± 0.1%	95.8 ± 0.1%	90.4 ± 0.1%	90.9 ± 0.1%	44.0 ± 0.1%
**Graph Neural networks**					
LINE (Tang et al., 2015)	96.5 ± 0.1%	96.2 ± 0.2%	90.5 ± 0.2%	85.9 ± 0.3%	36.2 ± 0.4%
GAE (Tang et al., 2016)	93.7 ± 0.1%	81.3 ± 0.7%	91.7 ± 0.1%	90.0 ± 0.1%	35.8 ± 0.1%
SDNE (Wang et al., 2016)	93.5 ± 1.0%	94.4 ± 0.4%	91.1 ± 0.6%	88.4 ± 0.8%	37.8 ± 0.8%
**Our model**					
SeBioGraph	97.2 ± 0.5%	96.4 ± 0.6%	93.1 ± 0.3%	89.9 ± 0.6%	48.8 ± 0.7%
- Auxiliary	93.8 ± 0.5%	87.1 ± 0.5%	88.9 ± 0.3%	85.6 ± 0.4%	39.1 ± 0.5%

Generally, compared to traditional methods [e.g., LLE (Roweis and Saul, 2000), LE (Belkin and Niyogi, 2003), and GF (Ahmed et al., 2013)], the existing proposed approaches have greatly enhanced the performance of link prediction. Especially in the STITCH CPI dataset with large-scale aggregation and edges, our methods are more effective. These results demonstrate that our methods can improve prediction performance in various biological link prediction tasks. Based on these results, we made the following observations: First, we can see that SeBioGraph significantly enhances the final result, which shows that transferring knowledge from learned graphs is effective. Second, our SeBioGraph achieves the best on all five datasets, indicating the robustness of prototype-based graph neural networks, auxiliary biomedical graph and hierarchical biomedical graph representation gate. In addition, as a metric distance-based semi-supervised method, SeBioGraph outperforms other existing methods and on the other hand, it achieves better performance than non-supervised methods and supervised methods.

## Conclusion

In this paper, we propose a novel framework called SeBioGraph. Our method strengthens the effectiveness of semi-supervised node classification and link prediction on a new target biomedical graph through conducting knowledge transfer which is learned from auxiliary graphs. Built upon the semi-supervised deep learning, SeBioGraph joints graph-level and local node-level global knowledge to learn a transferable metric space characterized. The experimental results show our proposed model is effective for two-class biomedical graph tasks in eight datasets.

## Data Availability Statement

The original contributions presented in the study are included in the article/supplementary material, further inquiries can be directed to the corresponding author/s.

## Author Contributions

YM is responsible for designing the framework. QL is responsible for the writing of the paper and specific experiments. NH and LL are responsible for designing the framework and idea. All authors contributed to the article and approved the submitted version.

## Conflict of Interest

LL was employed by company China Construction Science & Technology Group Co., Ltd. The remaining authors declare that the research was conducted in the absence of any commercial or financial relationships that could be construed as a potential conflict of interest.
